# Health literacy at work – individual and organizational health literacy, health supporting leadership and employee wellbeing

**DOI:** 10.1186/s12913-023-09766-0

**Published:** 2023-07-06

**Authors:** Lara Lindert, Kyung-Eun (Anna) Choi, Holger Pfaff, Sabrina Zeike

**Affiliations:** 1 Faculty of Human Sciences, Institute of Medical Sociology, University of Cologne, Faculty of Medicine and University Hospital Cologne, Health Services Research, and Rehabilitation Science, Eupener Str. 129, 50933 Cologne, Germany; 2 Center for Health Services Research, Brandenburg Medical School Theodor Fontane, Fehrbelliner Str. 38, 16816 Neuruppin, Germany; 3 Health Services Research, MIAAI, Danube Private University (DPU) GmbH, Steiner Landstraße 124, 3500 12 Krems‑Stein, Austria; 4 Vivalue Health Consulting GmbH, Cologne, Germany

**Keywords:** Occupational health, Mental health, Employee health, Leadership behavior

## Abstract

**Background:**

Against the backdrop of demographic change and the shortage of skilled workers, employees’ psychological wellbeing is of special interest for employers. In previous studies, individual health literacy has already been positively associated with psychological wellbeing. However, in order to improve health literacy, it is essential to take into account both the individual prerequisites and the demands and complexity of the system in which individuals operate. As current studies primarily focus on employees’ individual health literacy and as the concept of organizational health literacy, so far, is mainly used in the context of health care institutions, this study investigates on the impact of organizational health literacy and health supporting leadership on the relationship between individual health literacy and employees’ psychological wellbeing in a big German company based in the financial sector.

**Methods:**

Data of an employee survey that was conducted in a big German company of the financial sector in October 2021 were analyzed by two mediation analyses using the PROCESS macro by Hayes for SPSS. A total of 2555 employees was included in analyses (51.4% male and 48.6% female).

**Results:**

The relationship between individual health literacy and employees’ psychological wellbeing is partially mediated by organizational health literacy, indirect effect *ab* 0.268 – CI [0.170, 0.378] and by health supporting leadership, indirect effect *ab* 0.228 – CI [0.137, 0.329].

**Conclusion:**

Study results provide new indications for planning and evaluating the health strategy of companies. Regarding the psychological wellbeing of employees, practitioners and researchers should focus not only on individual health literacy but also on organizational health literacy and health supporting leadership.

## Background

In Germany, employers are subject to the Occupational Health and Safety Act and are obliged to identify and prevent or mitigate possible risks to the mental health of their employees. In addition, they are obliged to offer support in the form of occupational integration management in the event of prolonged absence of employees [1]. However, it is not only against the legal background that employers should take care of the mental health of their employees. Particularly against the backdrop of demographic change and the shortage of skilled workers, it is becoming increasingly important for companies to employ workers as long as possible and in good health. Still, mental illness continues to be the second most reason for absence of work days [2]. For the mental health of employees, work plays a crucial role in two respects: in addition to positive effects such as salary and social participation, working life can also bring anxiety-provoking risk factors such as bullying, monitoring (, e.g. by managers), accident risks and health hazards, high performance demands, organizational changes, and job insecurity [3].

In recent years, the concept of health literacy has received increased scientific and political attention at national and international level [4, 5] and individual health literacy has already been associated positively with psychological well-being and negatively with depression in various studies [6–8]. Health literacy *“entails people’s knowledge, motivation and competences to access, understand, appraise, and apply health information in order to make judgments and take decisions in everyday life concerning healthcare, disease prevention and health promotion to maintain or improve quality of life during the life course”* [9]. Schaeffer et al. [10] and Pleasant et al. [11] point out that health literacy as a whole should be seen as an interplay at individual and organizational level: In order to improve health literacy, it is essential to take into account both the individual prerequisites and the demands and complexity of the system in which individuals operate [10]. Organizational health literacy *“is described as an organization-wide effort to transform organization and delivery of care and services to make it easier for people to navigate, understand, and use information and services to take care of their health”* [12]. The National Action Plan Health Literacy emphasizes the need to promote health literacy in all areas of life, including the work environment [4]. The fact that low health literacy is associated with negative health outcomes [13–16] and has been associated positively with psychological wellbeing and negatively with depression in different settings [6–8] is relevant for employers: the psychological wellbeing of employees is one of the key factors to a healthy and successful organization. Furthermore, a high level of health literacy can have a positive impact on employees' ability to work and reduce occupational hazards and injuries [17–19].

As there are already several studies on the association between individual health literacy and psychological wellbeing, research on organizational health literacy – especially in companies outside the health care sector – is rather rare [20]. Furthermore, managers play a key role in a humane organization of work [21] and – with regard to organizational health aspects – function as multipliers and role models for their employees: managers may not demand health-conscious behavior of their employees, when not acting as a good role model [22, 23]. Several studies already reveal positive associations between leadership behavior (, e.g. transformational leadership) and health-oriented leadership and psychological wellbeing of employees [21, 24–29].

Against this background, this study analyzes data from an employee survey in a big German company in the financial sector and examines whether organizational health literacy and health supporting leadership have an impact on the association between individual health literacy and psychological wellbeing of employees. Research questions are:

1. What influence does organizational health literacy have on the association between individual health literacy and employees’ psychological wellbeing?
2. What influence does health supporting leadership have on the association between individual health literacy and employees’ psychological wellbeing?

To our knowledge, this is the first study that examines the impact of organizational health literacy and health supporting leadership on the association between individual health literacy and psychological wellbeing of employees. According to Schaeffer et al. [10] and Pleasant et al. [11] and taking into account the associations of leadership behavior and employees’ psychological wellbeing, we assume that the association between individual health literacy and employees’ psychological wellbeing is mediated by organizational health literacy and health supporting leadership. Study results may help to derive new evidence for researchers and practitioners regarding health literacy aspects in the context of employees’ psychological wellbeing and underline the relevance of organizational health literacy outside the health care sector and the role of managers in this context.

## Methods

We used data of an employee survey in a big German company of the financial sector that was conducted in October 2021 over a four-week period and thus during the 4^th^ wave of the SARS-CoV-2 pandemic. The overall aim of the survey was to develop and collect new key performance indicators for controlling the occupational health management. The survey also served to steer the companies’ health strategy by operationalizing and surveying health literacy aspects. Secondly, the results of the survey form the basis for developing individual measures for specific target groups. The survey was aligned for employees of all company sites in Germany, who were affiliated to the company during survey period and who wished to participate voluntarily. All employees were invited and reminded to participate online or via paper-based questionnaires by the company’s human resource management. Also, managers were given the task of reminding their employees to participate in the survey. Data were provided anonymously for scientific research purpose. All participants have given their consent.

### Study population

A total of 4980 employees took part in the employee survey (response rate 41.51%). 2555 participants answered all items on psychological wellbeing, individual and organizational health literacy, health supporting leadership, age, and gender that were relevant to answer research question (study sample). In this study sample 51.4% of the participants assigned to male and 48.6% to female gender. 12.8% of study sample were ≤ 30 years, 18% between 31 and 40 years, 22.6% between 41 and 50 years, 38.8% between 51 and 60 years and 7.8 were over 60 years old (see Table 1).

**Table 1:** Descriptive of study sample

	N			%
Age				
2003≤ 30 years	328			12.84
31 to 40 years	459			17.96
41 to 50 years	578			22.62
51 to 60 years	990			38.75
> 60 years	200			7.83
Gender				
Male	1313			51.39
Female	1242			48.61
	N	M	SD	Median (min/max)
Psychological wellbeing	2555	13.81	5.31	15.00 (0.00/25.00)
Individual health literacy	2555	2.74	0.53	2.75 (1.00/4.00)
Organizational health literacy	2555	2.76	0.65	2.83 (1.00/4.00)
Health supporting leadership	2555	2.63	0.54	2.71 (1.00/4.00)

*N* Number of individuals in study population, *M* Mean value, *SD* Standard deviation

### Measures

In a scientific focus group meeting including psychologists and rehabilitation scientists, the project team defined a common understanding of health literacy against the backdrop of the employee survey’s aim. As available scales focus – for the aim of the employee survey – too little on health-promoting aspects and rather on providing and understanding of health information, the scales for employees’ individual health literacy, organizational health literacy and health supporting leadership were newly developed in German language and pretested with employees. Furthermore, the scales where formulated in a way that recommendations could be derived from the results of the employee survey directly. Individual health literacy was measured using eight items, e.g. “I plan my everyday life in such a way that I stay healthy”, “I have a plan that is practical for me and that suits me, with which I can achieve my health goals”, that could be answered on a four-point Likert scale (1 = “I do not agree at all” to 4 = “I totally agree”). In this case, individual health literacy aims at identifying to what extent employees are aware of health promoting behaviors and plan and integrate health promoting behaviors in their daily life, and corresponds to the definition of health literacy according to Sørensen et al. [9]. Cronbachs’ Alpha was 0.88. To operationalize organizational health literacy six items where developed that could be answered on a four-point Likert scale (1 = “I do not agree at all” to 4 = “I totally agree”). The aim of this scale was to reveal how employees perceive efforts of the company to provide sufficient health promotion offers, to support health promoting work and life style and to involve employees in operational changes processes, e.g. “I am convinced that the company provides its employees with sufficient health promotion offers”, “I have the impression that the company supports the health-promoting activities and ideas of its employees” or “I am convinced that the company sufficiently promotes the skills and abilities of employees to take charge of their own health”. The scale corresponds to the definition of organizational health literacy according to Farmanova et al. [12]. Cronbachs’ Alpha was 0.91. Health supporting leadership was operationalized by seven items that could be answered on a four-point Likert scale (1 = “I do not agree at all” to 4 = “I totally agree”) and aims at identifying supportive aspects of the leadership behavior in the context of employee health. All questions referred to the direct supervisor, e.g. “My direct manager is a positive role model in his/her health behaviour”, “My direct manager makes it clear through his/her actions that he/she cares about safety and health at work” or “If my health is not good or I have difficulties in doing my work, my direct manager notices this and initiates a conversation”. Cronbachs’ Alpha was 0.87. The employees’ psychological wellbeing was operationalized by the German version of the WHO (Five) Well-Being Index (WHO-5), that comprises five questions that could be answered on a six-point scale (0 = “never” to 5 = “the whole time”). The items focus on the feelings within the last two weeks, e.g. “Over the last two weeks I have felt cheerful and in good spirits” [30]. A raw value of 0 indicates the worst, a raw value of 25 indicates the best possible wellbeing. Values below 13 can be seen as indicator to test for depression [31]. Cronbachs’ Alpha in this study was 0.89. Age was categorized in ≤ 30 years, 31 to 40 years, 41 to 50 years, 51 to 60 years, and > 60 years. Participants could assign themselves to male, female, and diverse gender.

### Statistical analysis

Two mediation analyses were performed using the PROCESS macro by Hayes (2022, v4.1) [32] in SPSS 23. For total, direct, and indirect effects, PROCESS uses ordinary least squares regression, yielding unstandardized path coefficients. To compute the confidence intervals and inferential statistics, bootstrap inference for model coefficients was computed with 5000 bootstrap samples and heteroscedasticity-consistent inference was calculated according to Davidson & MacKinnon [33]. Effects were interpreted as significant when the confidence interval did not include zero. The significance values of indirect effects were calculated according to Sobel [34].

In model 1, the focus was on the impact of individual health literacy on psychological wellbeing of employees and whether the direct path would be mediated by organizational health literacy. In model 2, the impact of individual health literacy on psychological wellbeing and the mediating effect of health supporting leadership on the direct path were examined.

## Results

**Fig. 1:**
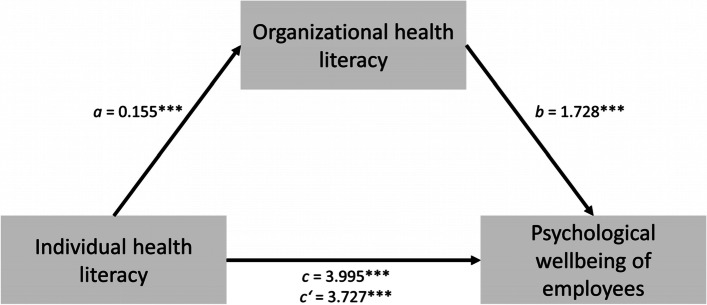
Mediation Model 1

**Fig. 2:**
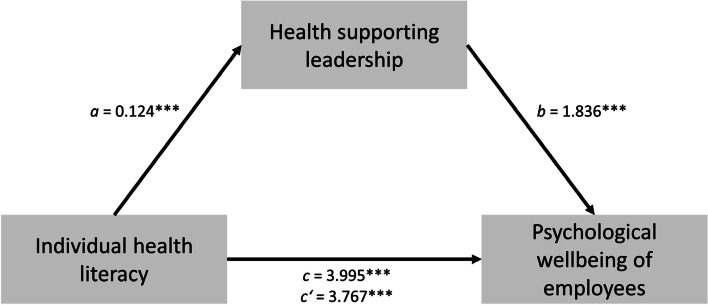
Mediation Model 2

In this study sample, the mean of psychological wellbeing was 13.81 (range 0 to 25), of individual health literacy 2.74 (range 1 to 4), of organizational health literacy 2.76 (range 1 to 4) and of health supporting leadership 2.63 (range 1 to 4) (see Table 1). The aim of the analyses was to examine whether organizational health literacy and health supporting leadership mediate the association between individual health literacy and employees’ psychological wellbeing. According to Schaeffer et al. [10] and Pleasant et al. [11] and taking into account the associations of leadership behavior and employees’ psychological wellbeing, it was assumed that the association between individual health literacy and employees’ psychological wellbeing is mediated by organizational health literacy (see Fig. 1 and Table 2) and health supporting leadership (see Fig. 2 and Table 3).

**Table 2:** Mediation Model of the effect of individual health literacy, organizational health literacy, and psychological wellbeing of employees

Dependent variable = psychological wellbeing	Coefficent of effect	SE	LLCI	ULCI
Direct effect of IHL on mediator OHL	0.155***	0.027	0.102	0.209
Direct effect of IHL on PW	3.727***	0.189	3.356	4.098
Direct effect of OHL on PW	1.728***	0.154	1.426	2.029
Indirect effect of IHL on PW	0.268***	0.053	0.170	0.378
Total effect on PW	3.995***	0.190	3.622	4.368

*SE* Standard error, *LCI* Lower level confidence interval, *ULCI* Upper level confidence interval, *IHL* Individual health literacy, *OHL* Organizational health literacy, *PW* Psychological wellbeing

^***^p < 0.001

**Table 3:** Mediation Model of the effect of individual health literacy, health supporting leadership, and psychological wellbeing of employees

Dependent variable = psychological wellbeing	Coefficent of effect	SE	LLCI	ULCI
Direct effect of IHL on mediator HSL	0.124***	0.023	0.080	0.170
Direct effect of IHL on PW	3.767***	0.187	3.400	4.134
Direct effect of HSL on PW	1.836***	0.190	1.464	2.209
Indirect effect of IHL on PW	0.228***	0.049	0.137	0.329
Total effect on PW	3.995***	0.190	3.622	4.368

*SE* Standard error, *LCI* Lower level confidence interval, *ULCI* Upper level confidence interval, *IHL* Individual health literacy, *HSL* Health supporting leadership, *PW* Psychological wellbeing

^***^p < 0.001

An effect of individual health literacy on psychological wellbeing was observed in both models, c = 3.995, *p* < 0.001 (see Figs. 1 and 2, and Tables 2 and 3). After entering organizational health literacy as mediator into model 1, individual health literacy predicted organizational health literacy significantly, *a* = 0.155, *p* < 0.001, which in turn significantly predicted psychological wellbeing, *b* = 1.728, *p* < 0.001. We found that the relationship between individual health literacy and psychological wellbeing of employees is partially mediated by organizational health literacy, indirect effect *ab* 0.268, *p* < 0.001 (see Fig. 1 and Table 2).

After entering health supporting leadership as mediator into model 1, individual health literacy predicted health supporting leadership significantly, *a* = 0.124, *p* < 0.001, which in turn significantly predicted psychological wellbeing, *b* = 1.836, *p* < 0.001. We found that the relationship between individual health literacy and psychological wellbeing of employees is partially mediated by health supporting leadership, indirect effect *ab* 0.228, *p* < 0.001 (see Fig. 2 and Table 3).

## Discussion

In summary, results indicate that individual health literacy, organizational health literacy and health supporting leadership are positively associated with employees’ psychological wellbeing and that organizational health literacy and health supporting leadership both partially mediate the association between individual health literacy and employees’ psychological wellbeing. The results are in line with previous studies on the relationship of individual health literacy with psychological wellbeing (and depression) [6–8] and on associations between health-oriented leadership and psychological wellbeing of employees [28, 29].

To our knowledge, this is the first study that examines the mediating impact of health supporting leadership on the relationship between individual health literacy and employees’ psychological wellbeing. Furthermore, to date, research on organizational health literacy – especially in companies outside the health care sector – is rather rare [20] and, to our knowledge, this is the first study that examines the impact of organizational health literacy on the association between individual health literacy and psychological wellbeing of employees. Current studies primarily focus on employees’ individual health literacy (also referred to as work-related health literacy or occupational health literacy) and interventions on individual (work-related / occupational) health literacy mainly focus on mental aspects [17]. So far, especially the concept of organizational health literacy is mainly used in the context of health care institutions (, e.g. hospitals, facilities for people with disabilities) and with regard to patient recipients [20]. However, Sørensen et al. [9] state that *“placing greater emphasis on heath literacy outside of healthcare settings has the potential to impact on preventative health and reduce pressures on health systems”* and Schaffer et al. [4] emphasize the promotion of health literacy in all areas of everyday life, including occupation and the workplace.

Study results highlight not only – in line with previous studies – the relevance of individual health literacy but also of organizational health literacy with regard to employee wellbeing and – or rather also – in institutions outside the health care sector. Based on our results, we support the recommendation of Schaeffer et al. [10] and Pleasant et al. [11], that individual and organizational health literacy should be seen as interplay. Taking into account the role of managers as multipliers and role models and the mediating effect of health supporting leadership in this study, we also recommend to additionally focus on health supporting leadership when it comes to health literacy in companies. In summary, researchers and practitioners should focus on various aspects and mechanisms when it comes to employees’ psychological wellbeing and health literacy. Furthermore, to improve health literacy in companies, Eickholt et. al [35] highlight the relevance of empowerment, organizational development and the setting approach (settings as living environments in which people spend a large part of their time – such as companies – and which (co-)determine their behavior).

Study results are of great relevance also facing the SARS-CoV-2 pandemic, as many workplaces have been relocated to home offices since 2020. On the one hand, home office demands a higher degree of self- and time management from employees: they must develop their own work structure and independently ensure that they keep to their working hours [36]. On the other hand, occupational health and safety is challenged, as measures originally established in the workplace reach employees in the home office less effectively or not at all [35, 37]. In addition, employees experience challenges with regard to organizational and management culture (negative image of home office, lack of trust on the part of managers and colleagues, high presence culture) [38]. Thus, both individual and organizational conditions (including the role of managers) must be created that enable healthy working in the home office and protect the mental health of employees. Regarding study results and addressing both individual and organizational health literacy might be a useful concept to face those challenges.

However, more research on the complex relationships between individual health literacy, organizational health literacy, employees’ psychological wellbeing and the important role of managers in this context is needed. Facing the increase of working from home as a result of the SARS-CoV-2 pandemic and the challenges this brings for employers, managers and employees, future health literacy research should as well focus on home office aspects.

### Strengths and limitations

As it was the task of managers to remind their employees regarding study participation, the recruitment might be biased. Managers who did not support the employee survey for various reasons, may have rather not remembered their employees to participate. Furthermore, the employee survey was conducted during the 4^th^ wave of the SARS-CoV-2 pandemic in October 2021. Therefore, SARS-CoV-2 specific aspects (, e.g. new ways of communication in home office, lack of trust from supervisors and colleagues, challenges in occupational health and safety management) may also play a crucial role regarding the research questions in this study. However, this needs to be further examined in future research, as those aspects were not covered in the employee survey of this company.

It also has to be considered, that, to date, definitions and instruments on individual/work-related health literacy differ between studies [17] and that organizational health literacy so far was mainly investigated in health care institutions and with patient recipients [20]. Therefore, study results are not comparable one-to-one with results of previous studies. Furthermore, the present results are subject to limitations, as the used scales with reference to health literacy and health supporting leadership have not yet been validated. However, this step was necessary to meet the specific needs and goals of the company and reflects the approach under real conditions. This points to possible limitations in the use of scientific (validated) scales when applied in practice, which remains to be discussed for the future.

## Conclusion

When it comes to employees’ psychological wellbeing not only individual health literacy but also organizational health literacy and health supporting leadership is of importance for practitioners (, e.g. in case of employee surveys and steering the company’s health strategy) and researchers. Future research should pay attention to the concept of (organizational) health literacy also outside of health care institutions and focus on the discussion and validation of instruments and on gathering longitudinal data to investigate causality.

## Data Availability

The dataset analyzed during the current study is not publicly available due to data protection reasons. Data are available from the corresponding author on reasonable request and with permission of the company, in which the employee survey was conducted.

## Abbreviations

WHO: World Health Organization
WHO-5: WHO (Five) Well-Being Index
